# NLP-based removal of personally identifiable information from Hungarian electronic health records

**DOI:** 10.3389/frai.2025.1585260

**Published:** 2025-05-30

**Authors:** András Berzi, Ervin Berényi, Zita Képes, Barnabás Antal, Ábrahám Gergely Varga, Miklós Emri

**Affiliations:** ^1^Division of Nuclear Medicine and Translational Imaging, Department of Medical Imaging, Faculty of Medicine, University of Debrecen, Debrecen, Hungary; ^2^Division of Radiology and Imaging Science, Department of Medical Imaging, Faculty of Medicine, University of Debrecen, Debrecen, Hungary; ^3^Organisational units directly managed by the Healthcare Vice-Chancellor, Health Finance Directorate, Chancellery, University of Debrecen, Debrecen, Hungary; ^4^Organisational units directly managed by the Healthcare Vice-Chancellor, Process Regulation Centre of the Clinical Centre, Chancellery, University of Debrecen, Debrecen, Hungary

**Keywords:** de-identification, electronic health records (EHR), General Data Protection Regulation (GDPR), Hungarian, low-resource language, named entity recognition (NER), natural language processing (NLP)

## Abstract

**Introduction:**

Electronic health records (EHR) in text format serve as crucial resources for data-driven medical research. To safeguard patient confidentiality, under the General Data Protection Regulation (GDPR), strict measures are required to ensure personal data is anonymized or pseudonymized to protect individual privacy. Natural language processing has consistently proven effective in automating the de-identification of sensitive information.

**Methods:**

We present spaCy models to recognize personally identifiable information (PII) from a wide range of free-text medical records written in Hungarian, a low-resource language. To develop this model, we compiled a corpus of clinical documents by annotating sensitive information within electronic health records sourced from the University of Debrecen. To simplify the annotation process, we pre-annotated the documents using a rule-based method. The corpora comprises over 15,000 documents and includes more than 90,000 instances of PII. We trained several models using this corpus and also developed a separate validation corpus to assess their performance.

**Results:**

The performance evaluation of the de-identification models on the developed corpora resulted in *F*_1_-scores ranging from 0.9697 to 0.9926. On the validation corpora, the *F*_1_-scores ranged from 0.9772 to 0.9867, demonstrating that the models can effectively handle previously unseen examples. Our risk analysis revealed that 99.67% of the sensitive information was successfully removed from the validation dataset.

**Discussion:**

The results indicate that similarly to other state-of-the-art systems our model is highly effective at identifying PII in clinical texts, guaranteeing that sensitive information in clinical documents can be protected without sacrificing the quality or usability of the data for research purposes. Despite these positive outcomes, several areas remain to be improved, such as the conduction of additional testing on diverse datasets, particularly those from different healthcare institutions. With ongoing refinements, these models have the potential to greatly enhance the efficiency of data de-identification processes, ensuring compliance with privacy regulations while promoting the secure sharing of medical data for scientific progress.

## 1 Introduction

Text-based patient medical records are essential resources for data-driven medical research and applications, providing valuable opportunities to improve patient care, guide clinical decisions, provide further data for researchers, and to drive the development of innovative treatments. These records contain a wide variety of detailed information, e.g., patient histories and details about diagnoses, treatments, or outcomes, which are sought-after information by domain experts but mostly available only in unstructured formats (Hossain et al., 2023).

In Hungary, similarly to several other countries, healthcare information systems (HISs) often lack structured medical records. Instead, patient information is stored in a free-text format that complicates data utilization for research and clinical decision-making. This study focuses on the University of Debrecen Healthcare Big Data System (UDBD Health) which is a comprehensive data warehouse that archives medical data from 1.3 million patients. UDBD Health is designed as a hybrid system that integrates a local data lake with a data warehouse based on MS Azure Databricks. This architecture aims to support robust data management while complying with stringent data protection regulations.

Nevertheless, the sensitive nature of these records presents substantial privacy challenges. Personal information, including names, addresses, and other sensitive identifiers must be carefully protected to uphold the patients’ confidentiality. Regulatory frameworks, such as the General Data Protection Regulation (GDPR 2016/679) in the EU (EUR-Lex, n.d.), impose strict requirements to preserve the identities of individuals. These regulations require that PIIs must be obfuscated in clinical documents before being utilized for other purposes, for example research, where the identity of individual patients is irrelevant.

Obfuscation in this context refers to either anonymization or pseudonymization, depending on the type of data and the purpose of data processing. The distinction is particularly relevant under the GDPR, which mandates different levels of protection and compliance obligations for anonymized and pseudonymized data. While anonymization results in irreversible data transformation where re-identification is impossible, pseudonymization allows for re-identification under controlled conditions, thus remaining within the scope of GDPR requirements.

To ensure the lawful and secure processing of sensitive medical data, the University of Debrecen has implemented multiple layers of regulatory and procedural safeguards beyond the mandatory national legislation. These include internal regulations such as the Health Data Management and Data Security Policy and the Policy on Health Data Assets that establish comprehensive data protection principles, risk management protocols, and ethical as well as legal data usage guidelines. These policies govern data stored within the UDBD Health, a repository that facilitates large-scale medical research while maintaining strict data security measures.

Based on the privacy principles of GDPR by design and default, our university has conducted a Data Protection Impact Assessment (DPIA) to assess and mitigate potential risks associated with processing personally identifiable information (PII) in clinical texts. The DPIA evaluates the impact of text mining-based PII removal solutions on data subjects’ rights and freedoms, ensuring compliance with Articles 5, 6, 9, and 32 of the GDPR, particularly regarding lawful processing, data minimization, and security safeguards.

Furthermore, as part of this research initiative, the UDBD Health Research Data Repository Use and Operation Policy has been established to provide structured governance on accessing and utilizing de-identified data. This policy aligns with GDPR requirements for secondary data processing, assuring that de-identified datasets used for research purposes are handled with appropriate safeguards, including encryption, controlled access, and audit mechanisms.

Given that medical texts often not only contain patient-specific identifiers (e.g., name, address, and social security number) but also physician-related identifiers (e.g., name and license number), the approach of the current study provides a more comprehensive de-identification process compared to rule-based methods. The manual de-identification of large volumes of medical documents is a labor-intensive and a time-consuming process that is prone to errors (Douglass et al., 2004). Consequently, there is an increasing demand for automated solutions that can accurately detect and remove personally identifiable information from clinical texts. Finding this information in unstructured texts requires a specific natural language processing (NLP) task called named entity recognition (NER) that is capable to automatically identify and categorize named entities into previously defined groups.

The established NER models have shown great success, with *F*-scores over 0.95, in identifying sensitive information in clinical documents in different languages. This capability enables the fast and accurate de-identification of such documents (Yogarajan et al., 2020). In addition, leveraging NER models allows for the achievement of a process that is not only more efficient and cost-effective but also scalable and capable of efficiently handling the increasing volumes of data produced by modern healthcare systems.

In this study we aimed to develop a NER model for Hungarian, a low-resource language (Jelencsik-Mátyus et al., 2023), medical record PIIs. As such languages often lack extensive annotated datasets required to train robust models, de-identification in these settings seems incredibly challenging. Furthermore, clinical documents frequently contain uniquely named entities specific to a given country or an institution, which make texts even more complex. To address our internal issues in the UDBD Health Research Data Repository, we created a domain-specific corpus, and a combination of rule-based and machine-learning approaches were employed to develop a high-performing solutions.

## 2 Materials and methods

We utilized the spaCy NLP framework to create de-identification NER models to find personally identifiable information (PII) in clinical documents. The model was tailored to process large amounts of free-text medical records efficiently. Its foundation was a carefully curated corpus of electronic health records (EHR) sourced from the University of Debrecen using the UDBD-Health data warehouse.

### 2.1 Data

To collect data for the corpora, we used the UDBD-Health data warehouse, which incorporates medical data from multiple sources, one of these sources being EHRs, used in the University of Debrecen, which contains millions of unprocessed clinical documents. In order to gather a diverse range of examples that represent these documents thoroughly, we conducted an exploratory data analysis (EDA) to identify the most important document types containing sensitive information. During the collection process, the main emphasis was on the documents that contained clinician names, which was the most frequently occurring and one of the most sensitive pieces of information. In addition, these documents often include other categories of sensitive information as well. The texts belonging to each type were collected randomized to avoid retrieving the data sorted based on any identifier or other information in the table, such as the date.

### 2.2 Annotation

**Figure 1:**
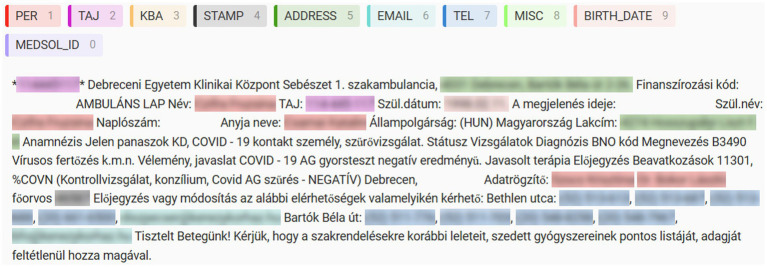
Label Studio annotation example.

Before annotating, the collected texts were pre-annotated with regular expressions (regex). Pre-annotating the documents could lead to a better quality final result, both in terms of accuracy and reliability. It can also help minimize the time required to process the documents (Fort and Sagot, 2010). The pre-annotated documents were manually corrected using Label Studio, an open-source data labeling platform (Label Studio, n.d.). The first half of the documents were checked and corrected by two annotators, while the remaining ones were reviewed by only one annotator. After the manual correction an automated test was performed to ensure that all entities were annotated (see Figure 1).

We defined 10 categories for the named entities, that fully represent the identified PIIs in the available data. The annotation process identified the following number of entities in each category (see Table 1):

**Table 1:** Table of training corpora entities.

Label name	Label description	Label count
PER	Doctor names, patient names, relative names, …	57,977
TAJ	Social security number	3,727
KBA	Central patient ID	1,743
STAMP	Doctor stamp number	5,887
ADDRESS	Address	5,275
EMAIL	Email address	1,529
TEL	Telephone number	7,991
MISC	Identity card numbers, account numbers, other specific IDs	1,317
BIRTH_DATE	Date of birth	3,427
MEDSOL_ID	EHR system specific user ID	747

**Figure 2:**
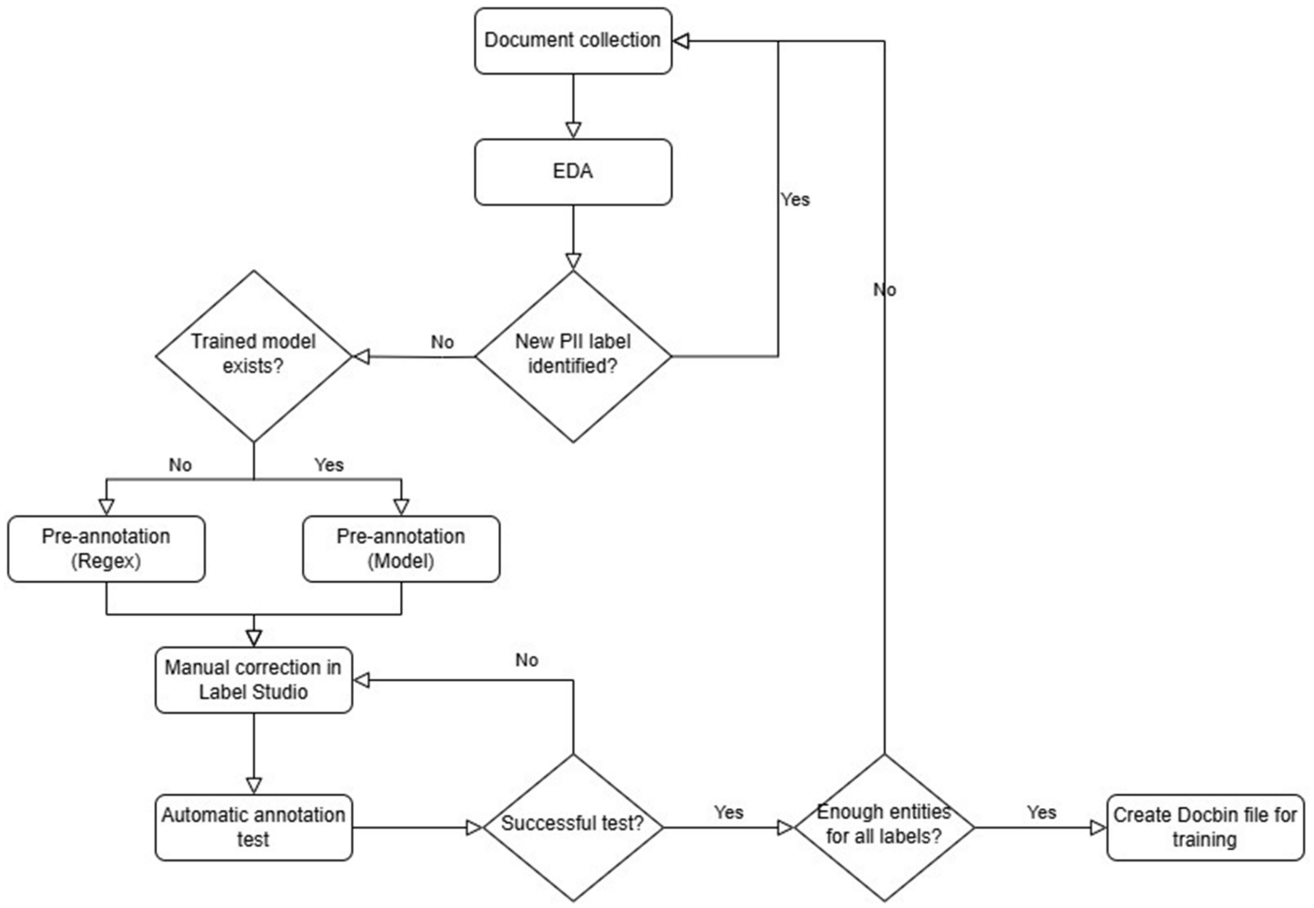
Flow diagram of annotation process.

The validation corpora were created after training the models, using the best-performing model on the test dataset for pre-annotation, resulting in a more accurate pre-processed dataset, which made the manual correction more effective. The validation corpora include document types used for the model training and texts from other document types, which were not included in the training dataset. Figure 2 shows the flow diagram of the annotation process.

### 2.3 spaCy

spaCy (spaCy, n.d.) is a free, open-source Python library for advanced natural language processing (NLP), specifically designed for production use, processing large amounts of text. Compared to other NLP frameworks, spaCy is preferred for real-world applications where speed, accuracy, and scalability are pivotal (Olabiyi et al., 2024). The framework offers both CPU and GPU-optimized pipelines, providing different types of model architectures and workflow optimization for specific requirements. Model architectures can be customized by changing or implementing custom components using PyTorch or TensorFlow. With spaCy-Transformers (spaCy, n.d.), it seamlessly integrates state-of-the-art transformer architectures and supports all available models from the HuggingFace library, which has pre-trained weights and a PyTorch implementation. SpaCy transformer models can process longer documents by dividing the input text into manageable slices before passing them through the transformer.

### 2.4 Training

We used two different transition-based (Lample et al., 2016) NER architecture configurations, where we experimented with different encoders, one using a BiLSTM encoder optimized for CPU and a Transformer model best performing with GPU. SpaCy provides a configuration file containing all the model’s settings and hyperparameters, enabling customization of the training process and ensuring consistent reproduction across various environments or experiments. The BiLSTM models were trained on a workstation equipped with 32 virtual CPUs and 64 GiB of memory, while Transformer models required more computational resources, including an NVIDIA A100 PCIe GPU. For the train-test split, we experimented with multiple datasets, including 80-20 and 90-10 splits, and additionally, a training dataset, where all the training data was used to test the model. The low amount of annotated examples for specific label categories influenced the choice of this training approach, e.g., MEDSOL_ID, MISC (see Table 2).

**Table 2:** Varieties of models.

Model name	Description
80_20_bilstm	No pre-trained components with BiLSTM encoder, trained on 80-20 train-test split
90_10_bilstm	No pre-trained components with BiLSTM encoder, trained on 90-10 train-test split
100_bilstm	No pre-trained components with BiLSTM encoder, no train-test split
90_10_bert-multi-uncased	Pre-trained google-bert/bert-base-multilingual-uncased (Devlin et al., 2019) model was used for transformer component, trained on 90-10 train-test split
90_10_bert-multi-cased	Pre-trained google-bert/bert-base-multilingual-cased (Devlin et al., 2019) model was used for transformer component, trained on 90-10 train-test split
90_10_hubert	Pre-trained SZTAKI-HLT/hubert-base-cc (Nemeskey, 2020; Nemeskey, 2021) model was used for transformer component, trained on 90-10 train-test split

### 2.5 Evaluation

The model’s performances were evaluated by *F*-score, Precision and Recall metrics traditionally used for NER models (Sokolova et al., 2006). Precision represents the proportion of entities returned by the model that are accurately identified, while Recall measures the proportion of relevant entities that were found. Individually, both metrics have their limitations, making it common practice to combine these two metrics using their weighted harmonic mean, known as the *F*-score. True positive (TP) indicates correctly found entities, false positive (FP) refers to incorrectly identified entities, and false negative (FN) represents missed entities. *F*-score is evenly balanced when *β* = 1 (*F*_1_-score), prioritizes Precision when *β* > 1, and favors Recall when *β* < 1. For the evaluation of our models, the *F*_1_-score was used.


$$\text{Precision}=\frac{\mathrm{TP}}{\mathrm{TP}+\mathrm{FP}}$$

$$\text{Recall}=\frac{\mathrm{TP}}{\mathrm{TP}+\mathrm{FN}}$$

$$F-\text{score}=\frac{(\beta^{2}+1)*\text{Precision}*\text{Recall}}{\beta^{2}*\text{Precision}+\text{Recall}}$$

$$F_{1}-\text{score}=\frac{2*\text{Precision}*\text{Recall}}{\text{Precision}+\text{Recall}}$$

## 3 Results

### 3.1 Training process

**Figure 3:**
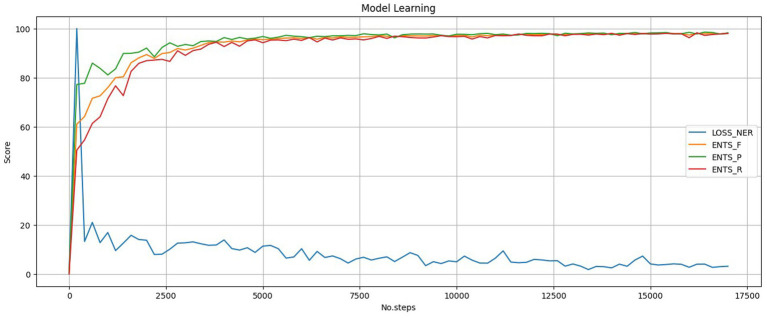
NER training result example.

For visualization, shown in Figure 3, we applied the MinMaxScaler to normalize the model metrics and the NER loss, scaling them to a consistent range. The model’s performance improved rapidly during the first 5,000 steps and improved in the next 10,000 steps, reflected by the increasing Precision, Recall, and *F*_1_-score metrics. At the same time, the NER loss decreased, indicating that the model was efficiently learning to predict the expected named entities. This trend was consistent across all the model training processes. For stopping criteria, we used the default settings from the spaCy configuration file, utilizing a patience parameter, which determines the number of steps to wait for performance improvement before terminating the training process, controlling early-stopping. The evaluation frequency was set to 200, and the patience was 1,600, meaning the model’s performance was evaluated every 200 steps, and training would stop if no significant improvement were made for 1,600 steps, while the maximum steps parameter was configured to 20,000; however, this upper limit was never reached.

### 3.2 Encoder test

**Figure 4:**
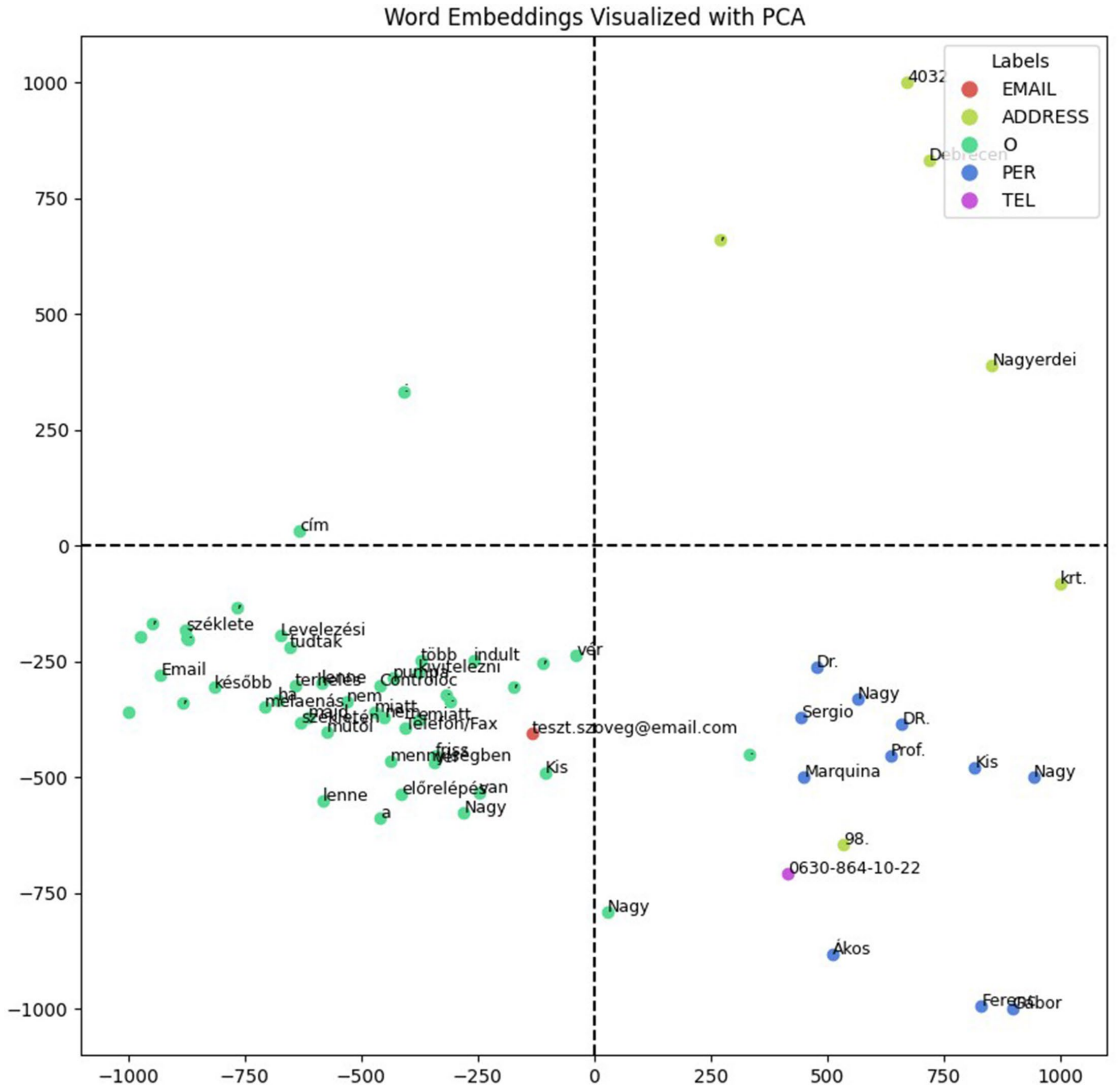
90_10_bilstm word embeddings with fictitious data.

**Figure 5:**
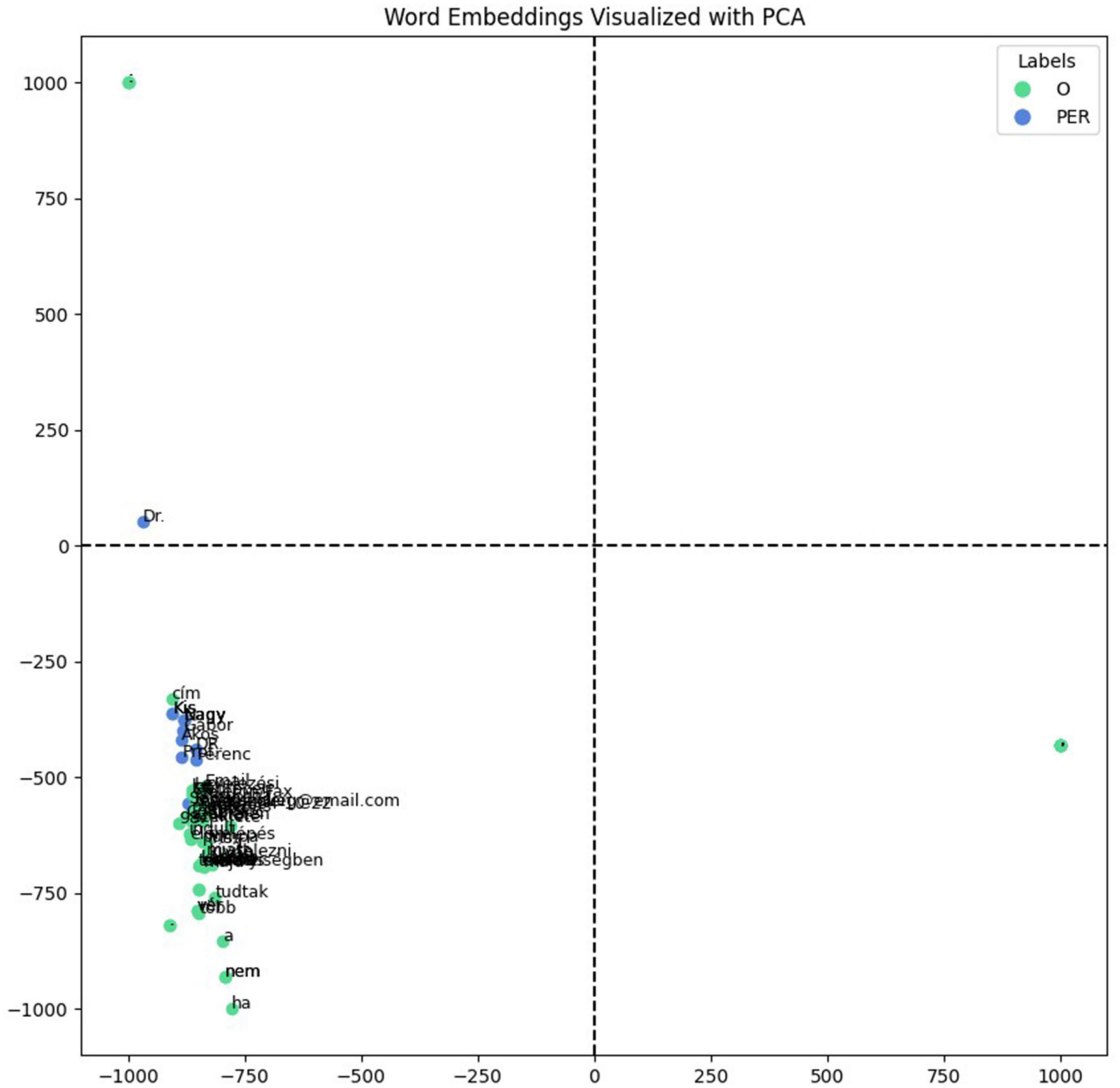
hu_core_news_lg word embeddings with fictitious data.

To evaluate and visualize the encoder, we created a test document containing fictitious data (names, email address, telephone number and address) with different types of named entities, including ambiguous examples that could be interpreted as both names and words with other alternative meanings. We compared one of our models, 90_10_bilstm, with another model, hu_core_news_lg (Orosz et al., 2022), a Hungarian CNN-based model, which provides named entity recognition and ships with pre-trained vectors. Both models successfully identified the names in the test document; however, only our model could extract specific named entities such as addresses, email addresses, and telephone numbers. For the visualizations, we extracted the vector representations of the tokens for each model and then projected them into a lower-dimensional space using principal component analysis (Karl Pearson, 1901) (PCA), enabling visualization in a 2D plot. The visualizations provided insights into the encoder’s learned representations, revealing patterns such as clustering the semantically similar entities. With our model’s representations, shown in Figure 4, the named entities predominantly grouped on the positive side of the x-axis, while the hu_core_news_lg model’s, shown in Figure 5, did not reveal distinct clustering patterns significantly. The spatial distribution highlights our model encoder’s ability to separate named entities from non-entity tokens effectively and suggests that the encoder learned the meaningful representations, capturing the semantic and systematic features necessary for distinguishing named entities in documents.

### 3.3 Training

**Figure 6:**
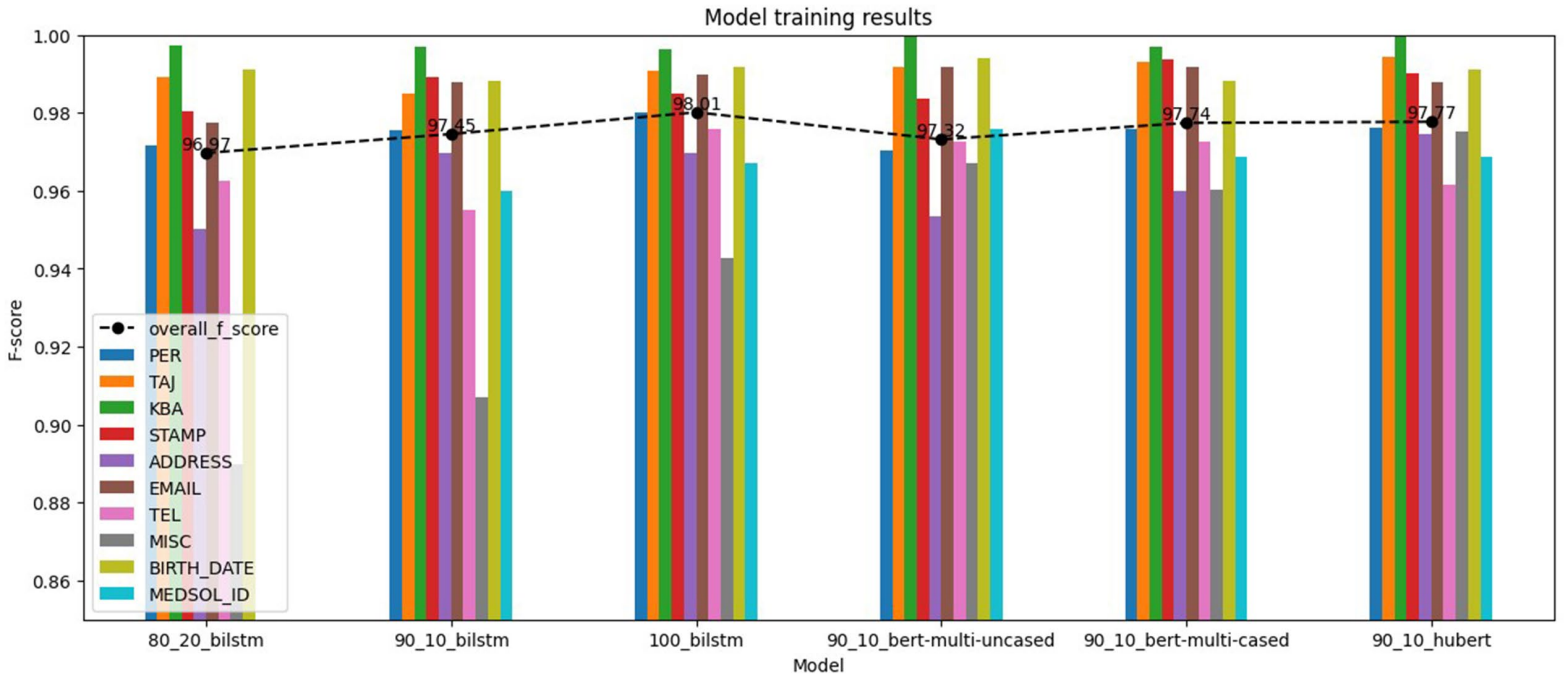
NER models training results.

Training results, shown in Figure 6, indicate the effectiveness of the NER models. All models achieved overall *F*_1_-scores over 0.9697 and individual label *F*_1_-scores over 0.84 on their test datasets. Using BiLSTM or Transformer architectures seems marginal, whereas different train-test splits make a more significant impact. Transformer models show improvement when utilizing language-specific models or increasing the number of parameters. In addition, the *F*_1_-scores of the labels in Transformer models appear to be more balanced, attaining higher *F*_1_-scores on the lowest-performing individual labels.

### 3.4 Validation

**Figure 7:**
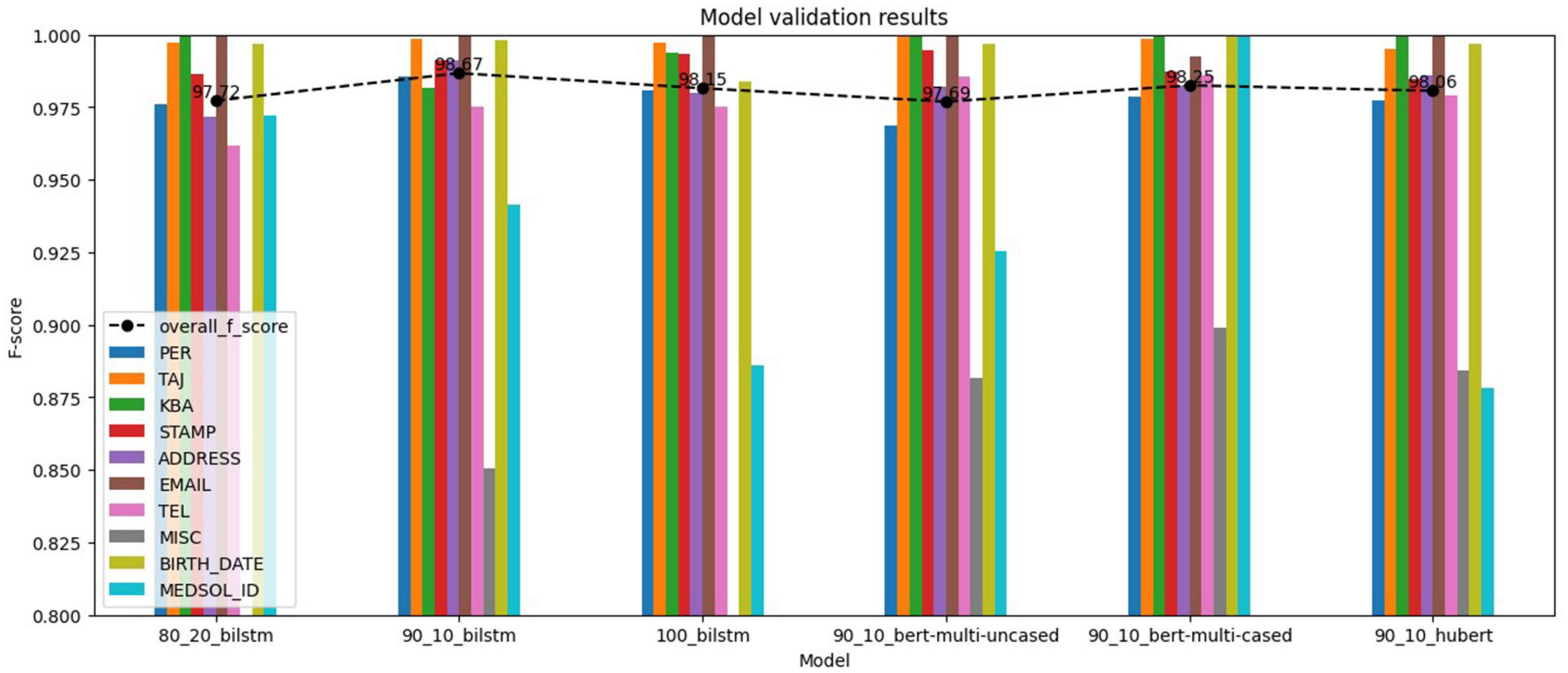
NER models validation results.

The evaluation results, shown in Figure 7, demonstrate even higher overall *F*_1_-scores compared to the training results, the lowest being 0.9769. On the other hand, the lowest label *F*_1_-score is 0.7253, significantly lower than we observed in the training results. This outcome could be attributed to either the limited number of annotated examples available for training or the complexity of the label type. We can also see that the differences remain marginal, but the top results come from other models.

### 3.5 Risk analysis

**Figure 8:**
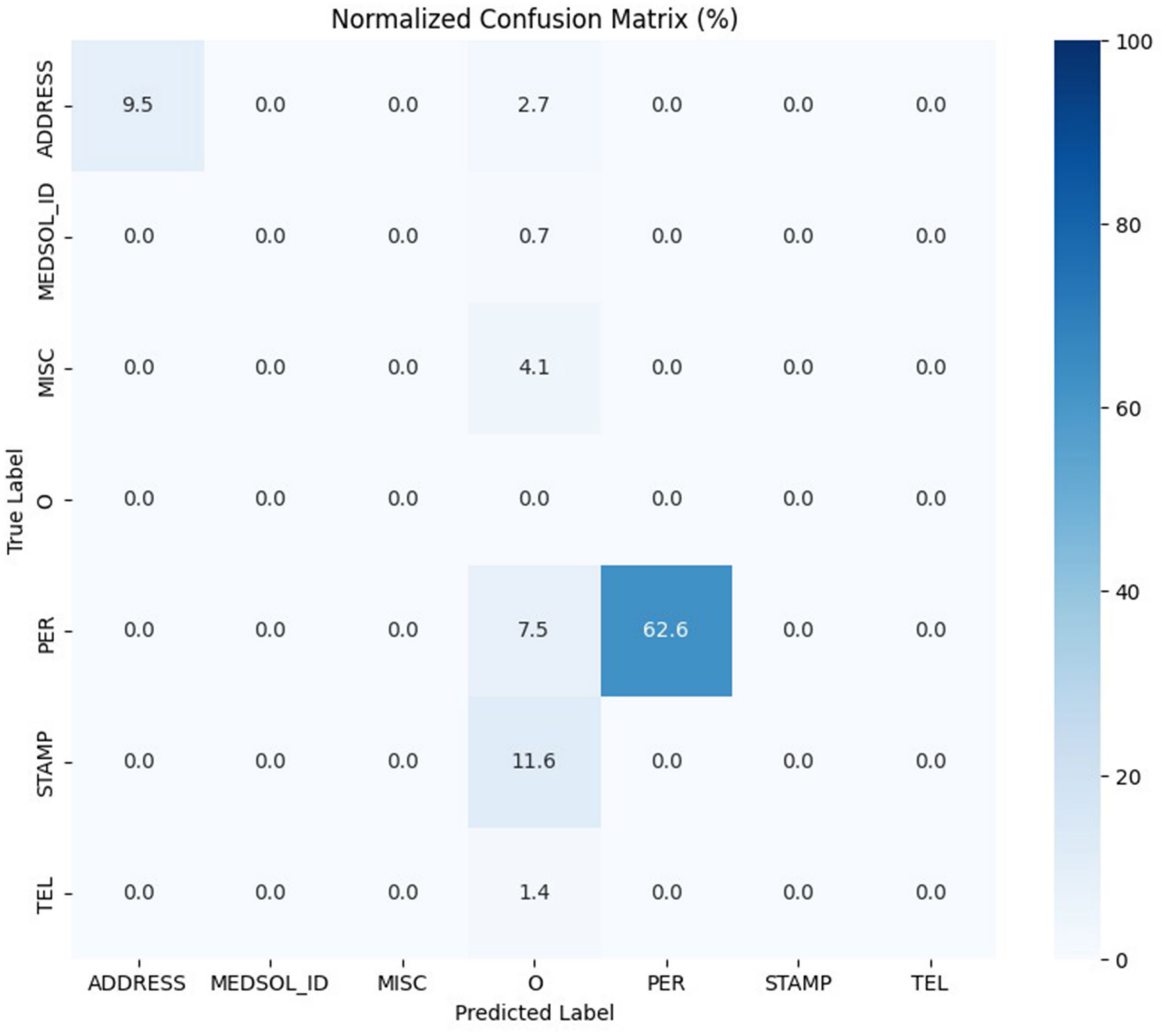
Normalized confusion matrix of the second de-identification process result.

We conducted a risk analysis on the validation dataset for one of our best-performing models, the 90_10_bilstm. First, we de-identified the validation dataset’s documents and replaced the found entities with a “<DEID>” token. After the de-identification, we pre-annotated and manually corrected the documents. The validation dataset originally contained 12,472 entities; after the de-identification process, this number was reduced to 147, which means that only 1.1786% of the PIIs remained in the documents. We found that repeating the de-identification process can further decrease the number of PIIs in our validation dataset due to the grammatical and spelling mistakes in the documents, which is a common characteristic of free-text medical documents. The remaining PIIs were reduced to 0.3287% (41) of the PIIs from the original documents. The distribution of the second de-identification process result is shown in Figure 8.

Based on the additional analysis performed on the remaining PIIs after the second de-identification we found that most of them were partial entities. The majority of these personal entities were for example forenames, which can be easily removed using rule-based methods. Nevertheless, limited numbers of single forename examples included in the training dataset could be attributable for the single left in the document. The other remaining entities derive from such document types, that could be completely excluded from our research data warehouse as they do not contain useful information for researchers.

### 3.6 Inference time and cost

**Figure 9:**
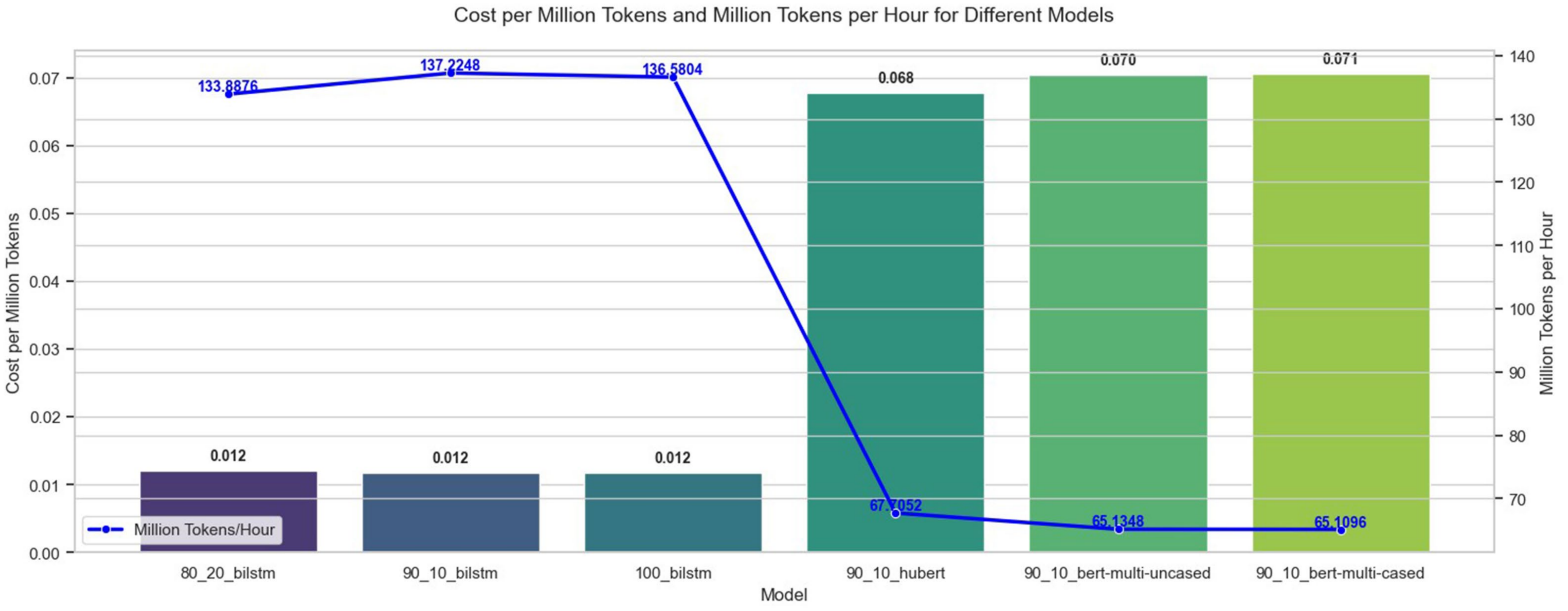
Inference times and costs of NER models.

Inference time and cost are critical considerations when deploying NER models in applications requiring large-scale text analysis. We evaluated our NER models with the same hardware configurations used for training and calculated inference costs using the prices of similar Azure VM instances based on the official pricing from the Microsoft Azure website (Microsoft, n.d.). Our inference results, shown in Figure 9, demonstrate that the BiLSTM models are significantly faster, inferring over 130 million tokens/h, and more cost-effective, costing 0.012 dollars to infer 1 million tokens, compared to Transformer-based models, which infer 65 million tokens/h and cost 0.07 dollars/1 million tokens. BiLSTM architectures achieve lower latency and require fewer computational resources, such as the ability to run effectively on CPUs, making them a practical choice for applications where real-time processing and cost efficiency are critical. In contrast, Transformer models can achieve more balanced and higher accuracy results while incurring substantially higher costs and longer processing times due to their larger parameter sizes. These findings highlight the importance of balancing performance and efficiency when selecting an NER model for deployment.

## 4 Conclusion

In the present study, we created the first annotated Hungarian corpora comprising de-identified clinical-related data. Using this database, we trained NER models with different architectures, achieving *F*_1_-scores similar to current state-of-the-art results (Kovačević et al., 2024). These models allow for reusing EHRs for secondary purposes by de-identifying PII while preserving valuable information.

Although, one potential limitation is that the models were trained on documents from a single institution, that could lead to restricted generalization on documents from other institutions, which is outside the scope of this study and mainly depends on the collaborative extension of the UDBD Health project. Additional testing with documents from other HISs would provide more information about the capabilities of this model. In future collaborations our workflow, models and experience gained during the development could be used to test, fine-tune or create more robust PII elimination models with better generalization capabilities, that can be used in other Hungarian HISs. Another limitation could be the size and the diversity of the corpora, which was narrowed down to a set number of document types. Using more types of documents for training could improve results for label categories that were underrepresented in the corpora and make other categories more robust.

The established NER models can efficiently and accurately identify most PII in Hungarian clinical documents from the Clinical Center of the University of Debrecen. Due to spaCy’s integration abilities the developed models are production-ready and easy to deploy allowing seamless implementation into our existing ETL pipeline, which pseudonymize clinical documents. In addition, there are further opportunities for development with more training data, that can be annotated using the created models with minimal need for manual correction.

Followed by implementing the evaluated PII-elimination method in the UDBD Health system’s data integration pipeline, we plan to focus on its post-training validation. To expand the annotated database records for re-training, human annotators will be asked to check the auto-de-identified parts of the medical reports, that will hopefully increase the accuracy of the model.

In our risk analysis we displayed that the analysis of residual PIIs makes pattern identification, the development of rule-based methods or the removal of entire documents with specific document types possible. To ensure sustained applicability, we are working on a model deployment strategy, which will include continuous risk assessments and mechanisms for incorporating regulatory updates.

Overall, this project represents a GDPR-compliant approach to de-identify free-text medical records in Hungarian, balancing the need for robust data protection with the facilitation of valuable medical research. The ongoing implementation of ethical approval processes and regulatory oversight ensures that all data processing activities remain within the framework of both Hungarian and EU data protection laws.

## Data Availability

The datasets presented in this article are not readily available due to patient confidentiality and privacy regulations. Requests to access the datasets should be directed to berzi.andras@med.unideb.hu.
